# Intrafraction motion during radiotherapy of breast tumor, breast tumor bed, and individual axillary lymph nodes on cine magnetic resonance imaging

**DOI:** 10.1016/j.phro.2022.06.015

**Published:** 2022-07-05

**Authors:** Maureen L Groot Koerkamp, H.J.G. Desirée van den Bongard, Marielle E.P. Philippens, Femke van der Leij, Stefano Mandija, Antonetta C Houweling

**Affiliations:** a Department of Radiotherapy, UMC Utrecht, Heidelberglaan 100, 3584CX Utrecht, The Netherlands; b Department of Radiotherapy, Amsterdam UMC, Meibergdreef 9, 1105 AZ, Amsterdam, The Netherlands; c Computational Imaging Group for MR Diagnostics & Therapy, Center for Image Sciences, UMC Utrecht, Heidelberglaan 100, 3584CX Utrecht, The Netherlands

**Keywords:** Accelerated partial breast irradiation, Regional lymph nodes, Intrafraction motion, Breast MRI, Supine, Prone

## Abstract

**Background and purpose:**

In (ultra-)hypofractionation, the contribution of intrafraction motion to treatment accuracy becomes increasingly important. Our purpose was to evaluate intrafraction motion and resulting geometric uncertainties for breast tumor (bed) and individual axillary lymph nodes, and to compare prone and supine position for the breast tumor (bed).

**Materials and methods:**

During 1–3 min of free breathing, we acquired transverse/sagittal interleaved 1.5 T cine magnetic resonance imaging (MRI) of the breast tumor (bed) in prone and supine position and coronal/sagittal cine MRI of individual axillary lymph nodes in supine position. A total of 31 prone and 23 supine breast cine MRI (in 23 women) and 52 lymph node cine MRI (in 24 women) were included. Maximum displacement, breathing amplitude, and drift were analyzed using deformable image registration. Geometric uncertainties were calculated for all displacements and for breathing motion only.

**Results:**

Median maximum displacements (range over the three orthogonal orientations) were 1.1–1.5 mm for the breast tumor (bed) in prone and 1.8–3.0 mm in supine position, and 2.2–2.4 mm for lymph nodes. Maximum displacements were significantly smaller in prone than in supine position, mainly due to smaller breathing amplitude: 0.6–0.9 mm in prone vs. 0.9–1.4 mm in supine. Systematic and random uncertainties were 0.1–0.4 mm in prone position and 0.2–0.8 mm in supine position for the tumor (bed), and 0.4–0.6 mm for the lymph nodes.

**Conclusion:**

Intrafraction motion of breast tumor (bed) and individual lymph nodes was small. Motion of the tumor (bed) was smaller in prone than in supine position.

## Highlights

• Intrafraction motion of the breast and individual axillary lymph nodes was studied.
• Displacements were investigated using cine magnetic resonance imaging.
• Motion was separated into breathing and drift components.
• Medians of the maximum displacements were small, <3 mm for breast and lymph nodes.
• Intrafraction motion of the tumor (bed) was less in prone than in supine position.

## Graphical abstract

**Figure:**
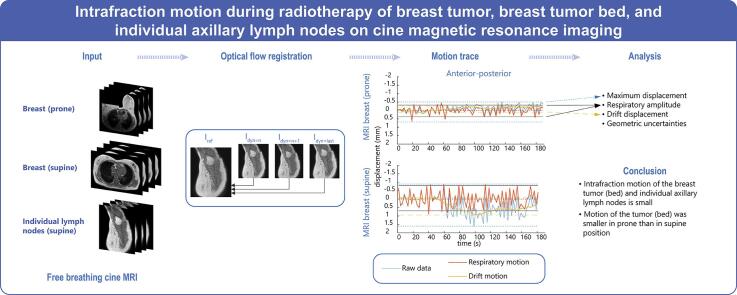

## 1 Introduction

Accelerated partial breast irradiation (APBI) is increasingly used to treat breast cancer patients, reducing the number of treatment fractions down to ten, five or even one [1], [2], [3], [4], [5], [6], [7]. With (ultra-)hypofractionation, the dose per treatment fraction and therefore delivery time increases. Consequently, the relative contribution of intrafraction motion to dose delivery uncertainties becomes increasingly important.

Position verification and correction after setup for APBI is usually performed using cone beam computed tomography. Displacements occurring after this initial correction are not corrected for and have to be considered in planning target volume (PTV) margins. Applying small, but adequate, margins is desirable to keep the irradiated volume as small as possible. Yet, what margin to apply is not well known for APBI.

To determine adequate margins, we need to quantify intrafraction displacements. Available studies on intrafraction motion required one or more fiducial markers [8], [9], [10] or used surrogate structures for motion tracking, such as the sternum or body surface [11], [12], [13], [14]. Also, often the temporal resolution of tracking, mostly using kV imaging or portal imaging, was limited. Cine magnetic resonance imaging (MRI) provides the opportunity to evaluate intrafraction motion of the target without the need of a surrogate tracking structure or fiducial markers and provides good temporal resolution, without additional imaging dose [15], [16], [17], [18].

Patients receiving APBI may be treated in prone or supine position. Few studies compared intrafraction motion between both positions, but only evaluated respiratory amplitude and not evolvement of displacement over time [14], [19], [20], [21]. For axillary lymph nodes, evaluations of intrafraction motion are limited to center-of-mass displacements of lymph nodes levels between different breathing phases or for deep-inspirational breath-hold [22], [23], [24], [25]; therefore they do not provide relevant measurements for boost treatment of individual lymph nodes.

In this study, we used cine MRI to evaluate intrafraction motion of the breast tumor or tumor bed and of individual axillary lymph nodes to determine the geometric uncertainties for (ultra-)hypofractionated treatment on either a conventional linac or an MR-Linac. For the breast, we compared prone and supine position.

## 2 Methods and materials

### 2.1 Patients

Between December 2016 and February 2020, 48 women with invasive breast cancer or ductal carcinoma in situ gave written informed consent for participation in this MRI study (Table 1). The study was approved by the institutional review board of the University Medical Center Utrecht (trial number NL56683.041.16). All women were referred to the radiotherapy department for preoperative and/or postoperative consultation for adjuvant radiotherapy. Thirty-three women participated prior to any treatment and 14 women participated during adjuvant radiotherapy. Twenty-three patients participated in the breast subgroup and 24 patients in the lymph nodes subgroup. One patient was not scanned because of anxiety.

**Table 1:** Patient characteristics.

	All (n = 47)	MRI breast (n = 23)	MRI lymph nodes (n = 24)
Age (median [range]) (years)	58 (26–78)	54 (26–72)	62 (42–78)
BMI (median [range]) (kg/m^2^)	25.6 (20.2–45.9)	24.7 (20.2–36.5)	25.8 (20.8–45.9)
Timing of MRI			
Preoperative	33 (69%)	14 (61%)	19 (76%)
Postoperative	15 (31%)	9 (39%)	6 (24%)
Tumor side•			
Left	25 (50%)	10 (43%)	15 (56%)
Right	25 (50%)	13 (57%)	12 (44%)
Tumor stage*			
T0	3 (6%)	1 (4%)	2 (7%)
Tis	7 (14%)	1 (4%)	6 (22%)
T1	24 (48%)	13 (57%)	11 (41%)
T2	15 (30%)	8 (35%)	7 (26%)
T3	1 (2%)	0 (0%)	1 (4%)
Nodal stage*			
N0	39 (78%)	17 (74%)	22 (81%)
N1	4 (8%)	3 (13%)	1 (4%)
N2	1 (2%)	1 (4%)	0 (0%)
N3	2 (4%)	0 (0%)	2 (7%)
Regional recurrence	1 (2%)	0 (0%)	1 (4%)
No SNB performed	3 (6%)	2 (9%)	1 (4%)

•Two patients had bilateral breast cancer.

*Tumor stage and nodal stage present cT and cN stage for preoperative patients and (y)pT and (y)pN stage for postoperative patients.

BMI: body mass index; SNB: sentinel node biopsy.

### 2.2 Patient positioning and imaging

All patients underwent 1.5 T MRI (Ingenia, Philips, the Netherlands). The breast subgroup was scanned in prone and supine position, except for one woman who was scanned only in supine position and two women who were scanned only in prone position. The lymph nodes subgroup was scanned only in supine position. More details on patient setup are described in our previous study [26]. In supine position, all women were positioned on the ThoraxSupport^TM^ (MacroMedics, The Netherlands) without inclination with both arms in abduction. A 16-elements anterior coil, mounted on coil bridges to prevent breast deformation, and the posterior coil integrated in the scanner table were used as receive coils. In prone position, the women were positioned on an in-house developed support (in collaboration with Orfit Industries, Belgium). The ipsilateral breast was hanging freely, and the contralateral breast was pulled aside. Two flexible surface coils, one on each side of the ipsilateral breast, and the posterior coil were used as receive coils. In the last 13 patients, the anterior coil was additionally placed on top of the patient when in-bore space was sufficient (8/13 patients).

All patients underwent two or three MRI sessions with repositioning in between, acquired on a single day (40 patients) or on different days (7 patients). Median duration of each session was 20 min (range: 7–35 min). In each session, a 1–3 min free-breathing cine MRI was acquired on average 15 min (range: 6–32 min) after start of imaging. We acquired 63 cine pairs of the breast and 63 cine pairs of the lymph nodes.

To investigate displacements along the three main axes, the cine MRI were acquired interleaved in two planes: transverse/sagittal for the breast subgroup and coronal/sagittal for the lymph nodes subgroup. The intersection between the planes was positioned through the breast tumor or tumor bed for the breast subgroup and through one axillary lymph node for the lymph nodes subgroup. This was planned on a 3D T1-weighted MRI acquired before the cine MRI. The cine MRI was acquired using a 2D gradient echo sequence, except for four cines that were acquired with a balanced steady-state free precession (SSFP) sequence. Scan parameters for the majority (>90%) of the cines were: repetition time/echo time = 2.8–2.9/1.4 ms, in-plane field-of-view = 350-450×350-500 mm^2^, slice thickness = 8 mm, flip angle = 10° (35° for balanced SSFP), acquired pixel size 1.8–2.0x1.8–2.0 mm^2^, reconstructed pixel size 1.6–1.7x1.6–1.7 mm^2^, sensitivity encoding of 1–1.2, and a scan interval of 0.6 s per cine, i.e. 1.2 s per orthogonal cine pair (one pair = coronal/sagittal or transverse/sagittal). For the remaining minority, all in the breast subgroup and mostly due to scanning challenges in prone position, the field-of-view was reduced down to 280x280 mm^2^, reconstructed voxels size ranged 1.1–2.0×1.1–2.0 mm^2^, and scan interval varied from 0.3 to 0.7 s. To get rid of the saturation band caused by acquisition of the orthogonal slices, occurring in every first slice acquired in a plane, all cines were acquired in reversed order for each second set of cine pairs, such that the acquisition order became transverse-sagittal-sagittal-transverse etc. for the breast cines and coronal-sagittal-sagittal-coronal etc. for the lymph node cines (Supplementary Material).

### 2.3 Image processing and motion quantification

**Fig. 1:**
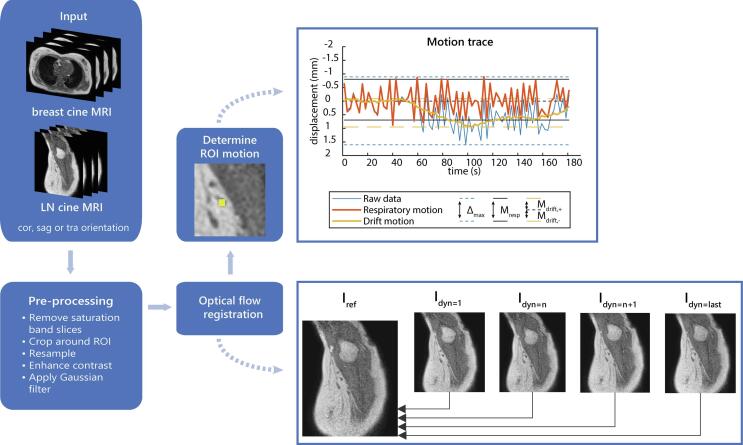
Schematic overview of the image registration and motion quantification steps. Abbreviations: cor: coronal, sag: sagittal, tra: transverse, dyn: dynamic slice, LN: lymph node, ROI: region of interest.

Because of the saturation band artefact that led to inaccurate registration results, we removed every other slice for each orientation from all cine MRI (Supplementary Material), which halved the temporal resolution. To improve registration results, image contrast was enhanced and the images were resampled to double resolution and smoothed to reduce noise (Fig. 1). In each cine MRI, we manually indicated a region of interest (ROI) in one reference slice to depict the breast tumor, tumor bed or lymph node to be tracked.

Next, all slices were registered to the reference slice using deformable registration with optical flow [27], [28], resulting in pixel-wise 2D deformation vector fields. Displacement of the target was quantified by taking the mean displacement vector of all ROI-pixels. From this, we calculated the maximum displacement (Δ_max_), respiratory motion amplitude (M_resp_), and drift displacement (M_drift_), see Fig. 1. Δ_max_ was defined as the distance between the extreme positions of the target. A moving average with a window of 30 s was used to calculate drift motion. The moving average was used to correct the raw motion signal to obtain the motion resulting from breathing. M_resp_ was calculated as the distance between the 5th and 95th percentiles of the breathing trace. The extreme positions in the drift motion were taken as M_drift_. All image processing and motion quantifications steps were performed using Matlab R2019a (The MathWorks, Inc., USA).

For the breast, we excluded 11 transverse and 9 sagittal cine series. For the lymph nodes, we excluded 10 coronal and 15 sagittal cine series. Exclusion reasons were (number of breast and lymph nodes cines respectively): the 2D image was not positioned correctly (2 and 4 cines), no clear tumor (bed) or lymph node visible (5 and 19 cines), an incorrect tracking result (13 and 2 cines).

### 2.4 Statistical analysis

Displacements were analyzed using descriptive statistics. For the subgroup of breast cine patients of whom we had both a supine and a prone cine MRI tracking result, we used a Wilcoxon signed-rank test to compare Δ_max_, M_resp_, and M_drift_ between prone and supine position (RStudio version 1.0.143, Rstudio Team, USA). In patients with multiple cine MRI of one position available, only the first acquired dataset was used for testing. A p-value < 0.05 was considered statistically significant.

### 2.5 Geometric uncertainties

To determine the geometric uncertainties resulting from the intrafraction motion, we calculated the mean and standard deviation of the raw motion traces. Subsequently, we calculated the mean of means (M), systematic error (Σ = standard deviation of means) and random error (σ = root mean squared of standard deviations).

Additionally, we calculated the uncertainty resulting from the breathing motion only. This σ_resp_ was calculated as the root mean square of 0.4*M_resp_ of all cines [10], [29].

## 3 Results

**Fig. 2:**
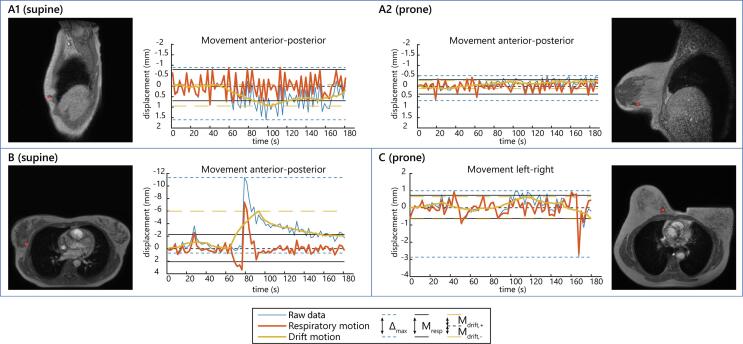
Example of motion traces in three different patients. A: sagittal cines of a patient with regular breathing motion in supine (A1) and prone (A2) position; B: transverse cine motion trace of a patient with a deep breath around 80 s in supine position, with a remaining drift in breathing level afterwards; C: transverse motion trace of a patient showing a single large motion peak around 165 s caused by bulk motion on the couch. Note that the y-axes are scaled differently for each patient.

**Fig. 3:**
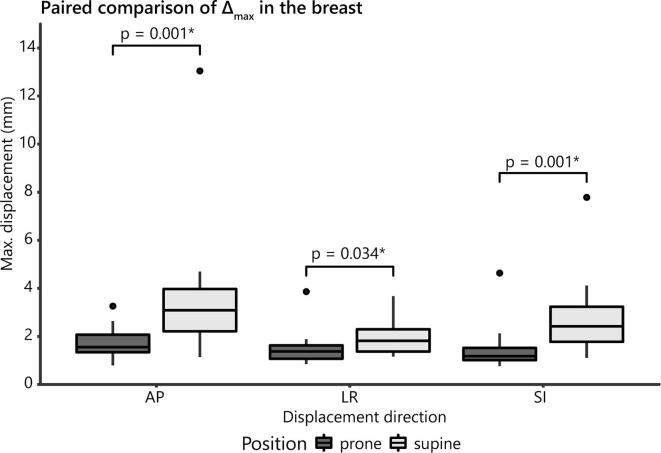
Comparison of Δ_max_ in prone vs. supine cine MRI acquired in the same patient. * = statistically significant.

Fig. 2 shows examples of motion traces of the breast tumor (bed) and illustrates larger displacements in supine than in prone position (video results in Supplementary Material). In general, Δ_max_, M_resp_ and M_drift_ were all larger in supine than in prone position, most obviously in anterior-posterior and superior-inferior direction (Table 2). M_drift_ was somewhat larger towards anterior and superior than towards posterior and inferior, especially in supine position. In the paired comparison, Δ_max_ was significantly larger in supine position (Fig. 3). M_resp_ was also significantly larger for supine position in anterior-posterior (p-value = 0.003) and superior-inferior direction (p-value < 0.001), but not in left–right direction (p-value = 0.105). M_drift_ was only significantly larger in supine position for the drift in the superior direction (M_drift,2_; p-value = 0.007).

**Table 2:** Motion of the breast tumor or tumor bed and individual axillary lymph nodes in cine MRI. Δ_max_ = maximum displacement; M_resp_ = respiratory amplitude; M_drift_ = maximum drift displacement. M_drift,1_ is towards the left (L), inferior (I) and posterior (P) direction; M_drift,2_ is towards the right (R), superior (S) and anterior (A) direction.

	**Parameter**	**LR**	**AP**	**SI**
		Median (range)	Median (range)	Median (range)
Breast (prone)	# cines	30	31	31
	Δ_max_ [mm]	1.3 (0.2–3.9)	1.5 (0.3–3.3)	1.1 (0.1–4.6)
	M_resp_ [mm]	0.8 (0.2–1.4)	0.9 (0.3–1.8)	0.6 (0.1–1.6)
	M_drift,1_ [mm]	0.2 (0–2.2)	0.2 (0–0.5)	0.1 (0–2.4)
	M_drift,2_ [mm]	0.1 (0–0.7)	0.3 (0–1.8)	0.2 (0–2.6)
Breast (supine)	# cines	22	23	23
	Δ_max_ [mm]	1.8 (0.8–4.2)	3.0 (1.1–13.1)	2.4 (1.1–7.8)
	M_resp_ [mm]	0.9 (0.4–2.4)	1.6 (0.6–4.1)	1.4 (0.7–2.6)
	M_drift,1_ [mm]	0.3 (0.0–1.0)	0.3 (0–2.0)	0.1 (0–1.5)
	M_drift,2_ [mm]	0.1 (0–1.3)	0.5 (0–5.3)	0.5 (0–3.3)
Lymph nodes (supine)	# cines	52	52	48
	Δ_max_ [mm]	2.2 (1.1–5.3)	2.4 (1.0–5.4)	2.3 (0.5–5.0)
	M_resp_ [mm]	1.4 (0.7–2.7)	1.5 (0.8–3.7)	1.4 (0.5–3.3)
	M_drift,1_ [mm]	0.2 (0–1.9)	0.1 (0–1.2)	0.2 (0–1.5)
	M_drift,2_ [mm]	0.2 (0–1.8)	0.3 (0–2.1)	0.3 (0–1.6)

The maximum displacements of individual lymph nodes were comparable in all directions with median values ≤ 2.4 mm (Table 2). Motion was mainly due to breathing, with median drifts in all directions of maximum 0.3 mm. The largest Δ_max_ were mostly caused by infrequent deep breaths, less often a drift occurred. In general, we observed a higher Δ_max_ for 3 min cine MRI than for 1 min cine MRI.

The systematic and random errors are shown in Table 3. Both uncertainties were below 0.4 mm in all directions for the breast in prone position. The uncertainties in supine position were larger and ranged up to 0.8 mm for the anterior-posterior direction. Systematic and random errors for the lymph nodes were similar in all directions, 0.4–0.6 mm.

**Table 3:** Geometric uncertainties for intrafraction motion on cine MRI. Mean of means (M), systematic error (Σ_total_), and random error (σ_total_) for all intrafraction motion and for breathing only (σ_resp_).

	**Parameter**	**LR**	**AP**	**SI**
Breast (prone)	M [mm]	0.0	−0.1	−0.1
	Σ_total_ [mm]	0.1	0.2	0.2
	σ_total_ [mm]	0.3	0.4	0.3
	σ_resp_ [mm]	0.3	0.4	0.3
				
Breast (supine)	M [mm]	0.1	−0.2	−0.2
	Σ_total_ [mm]	0.2	0.7	0.5
	σ_total_ [mm]	0.4	0.8	0.6
	σ_resp_ [mm]	0.5	0.8	0.6
				
Lymph nodes (supine)	M [mm]	−0.1	−0.0	−0.1
	Σ_total_ [mm]	0.4	0.4	0.4
	σ_total_ [mm]	0.6	0.6	0.6
	σ_resp_ [mm]	0.7	0.7	0.7

## 4 Discussion

Our results showed that intrafraction motion of the breast and axillary lymph nodes was small with median maximum displacements below 3 mm. Incidental large maximum displacements up to 13 mm were observed, but were mostly transient. Intrafraction displacements of the breast tumor (bed) were significantly smaller in prone than in supine position. In the analysis of separated drift and breathing motion, it was observed that this was mainly due to smaller breathing motion in prone position. The geometric uncertainties resulting from intrafraction displacement are small, at maximum 0.8 mm. Prone position may be favored over supine position for APBI or boost treatment because of the reduced intrafraction motion.

Intrafraction motion has been studied before in prone and supine position, though only few studies previously compared both positions [14], [19], [20], [21]. Using 4D computed tomography (CT) or magnetic sensors, these studies also showed smaller breathing amplitudes in prone position. Our results confirm the smaller respiratory motion in prone position, and are similar to the 0.5–1.1 mm for prone position [14], [20], [21], [30] and 0.6–3.0 mm for supine position [10], [14], [20], [21] earlier found. Our work contributes substantially by not only comparing breathing amplitude, but also displacement and trends over time between both positions in the same patient.

For both prone and supine position, the average displacements we observed were smaller compared to other studies [8], [9], [10], [11], [31]. Similarly, the systematic and random intrafraction errors in prone position were much smaller than the errors reported by Mouawad et al. and Lakosi et al. [8], [32]. Both the random and systematic intrafraction errors in supine position were also slightly smaller than those reported by Hoekstra et al., except for the systematic error in anterior-posterior which was comparable [10]. The difference may be explained by the longer durations over which displacements were quantified in the other studies.

The duration we evaluated is shorter than actual treatments –with 6–8 min beam-on time for single fraction (Volumetric-modulated arc therapy; unpublished data of [7]) or five-fraction (MR-Linac step-and-shoot intensity-modulated radiation therapy [26]) APBI– and is the most important limitation of this work as displacements were shown to increase with treatment duration [10]. Also, by acquiring the cine MRI on average 15 min after start of imaging, we may have missed the largest displacements, that were found to occur in the first minutes of a treatment fraction [13], [33]. However, when comparing to the time bin corresponding to the average time interval after which our cine MRI was acquired, our required margins for supine position would be comparable to the margins found by Hoekstra et al. for superior-inferior and anterior-posterior direction [10]. Contrarily, the 0.7 mm margin reported by Acharya et al. for longer durations is much smaller [31]. This was calculated by evaluating the margin necessary to cover at least 90% of the tumor bed for 90% of the time on cine MRI, which is a different evaluation approach and can therefore not be compared directly.

Our results showed that drift displacement, especially in supine position, was somewhat larger towards anterior and superior than towards posterior and inferior, which interestingly is opposite to the direction of the systematic drift found by others [10], [13], [33]. Most likely this difference is also related to the short time period evaluated, and could indicate that the observed largest drifts in our work are most likely due to deep breaths inducing larger displacements towards anterior and superior with respect to normal breathing. It should be noted that our drift results provide the maximum drift amplitude in each direction and not the average systematic drift.

This is the first study investigating motion of individual lymph nodes. Available studies only reported on displacements of lymph node levels [22], [23], [24], [25]. Our maximum displacements are similar to 0.2–2.2 mm center-of-mass displacements reported for axillary lymph node regions on 4D CT or comparison of exhale CT and normal breathing state CT [22], [23], which shows that the displacements we measured correspond to the magnitude of normal breathing motion. Much larger displacements of up to 14 mm in superior-inferior and 10 mm in anterior-posterior for different axillary lymph node levels were also reported [22], [24], [25], but these were found using deep-inspiration breath-hold CT. Even our largest maximum displacements of the lymph nodes, 5.4 mm, did not reach these values, showing that we did not observe very deep breaths. These results may be useful for stereotactic treatment of individual tumor-positive regional lymph nodes that cannot be surgically resected.

Another strength of our work is that by using MRI we could directly determine the displacement of the target instead of a surrogate such as a fiducial marker or body surface. Only few other studies reported on the use of (cine) MRI to investigate intrafraction motion of the breast [17], [30], [31]. The temporal resolution of cine MRI also allowed for studying the intrafraction motion on a scale of seconds, enabling distinction between drift and breathing motion.

Besides the limited duration of the cine MRI, this work has several other limitations. First, we had to discard imaging data because of the saturation band that impeded tracking. The remaining frequency in a single orientation (every 2.4 s) may have been too low to completely cover respiratory motion. Yet, we think that over the full cine duration the combination of breathing motion and drift is accurately captured in the motion trace statistics, by using the percentiles and average values, at least for the 3 min cine MRI. For the 1 min cine MRI, the motion may be underestimated by aliasing. Second, the displacements found are around or smaller than the voxel size. However, as the applied method allows for tracking sub-voxel displacements [34] and the image resolution is also the limit for manual tracking [27], we think the method was suitable to investigate breathing motion and drift. Third, the signal-to-noise ratio differed across the scans, this may have influenced the tracking accuracy. Fourth, the results could be influenced by out-of-plane plane motion. Taking into account the slice thickness and the maximum displacements observed, the impact is considered negligible. Additionally, simultaneous out-of-plane motion was captured with the interleaved orthogonal acquisition. Fifth, the majority of lymph nodes investigated were in level I, whereas lymph nodes requiring a boost are more often located in *peri*-clavicular levels that cannot be reached surgically. Higher level nodes were often not visible in patients with N0 stage. Finally, the visibility of individual lymph nodes was sometimes limited because of the small size and tumors or tumor beds were not always well distinguishable from surrounding breast tissue. In some patients we used biopsy marker or surgical clip artefacts as target structure in the cine.

This work shows that the intrafraction component of motion in the breast and for individual lymph nodes is limited and results in small geometric uncertainties. However, large structural drift or bulk motion was not observed in the evaluation. It should be evaluated if displacements in prone position remain smaller than in supine position for longer durations, as baseline drifts or patient relaxation will play a larger role in margins necessary for longer fraction durations. Future work should also address comparison of interfraction displacements for both positions to be able to evaluate impact on clinical application with respect to the current PTV margins. Additionally, future studies should investigate displacements of individual lymph nodes outside axillary level I. When applying smaller margins in hypofractionated treatments with increased duration, motion monitoring and motion mitigation techniques for non-transient large displacements should be considered, for instance by online MRI-guidance with gated dose delivery or target tracking strategies [31], [35], [36].

In conclusion, intrafraction displacements of the breast tumor or tumor bed and individual axillary lymph nodes on cine MRI were small. We showed that motion consisted of regular breathing motion and drift components and we did not observe lasting bulk displacements. The displacements and geometric uncertainties for the breast tumor (bed) in superior-inferior and anterior-posterior direction were smaller for prone than for supine position because of smaller breathing motion in prone position.

## Declaration of Competing Interest

The authors declare that they have no known competing financial interests or personal relationships that could have appeared to influence the work reported in this paper.
