# Supplementary material for: Intrafraction motion during radiotherapy of breast tumor, breast tumor bed, and individual axillary lymph nodes on cine magnetic resonance imaging
Source: Phys Imaging Radiat Oncol. 2022 Jul 5;23:74–9. doi: 10.1016/j.phro.2022.06.015 (PMC9271760; doi:10.1016/j.phro.2022.06.015)
Supplement: Supplementary data 5 [file mmc5.pdf]

Supplementary Material A – Interleaved cine MRI acquisition and saturation band

The interleaved acquisition of cine MRI in two orthogonal directions caused a saturation band in each direction after switching between slice orientations, which could obscure the breast tumor (bed) or lymph node (Figure S1, A&C, between blue arrow heads). To acquire images without the banding artefact, the acquisition order was reversed for each second cine pair such that two slices in the same orientation were acquired directly after each other. In each second slice in the same orientation the saturation band was not present anymore, and the tumor (bed) or lymph node is visible again (Figure S1, B&D, red arrow). Slices with a saturation band were removed and not included in the analysis, resulting in halving of the temporal resolution.

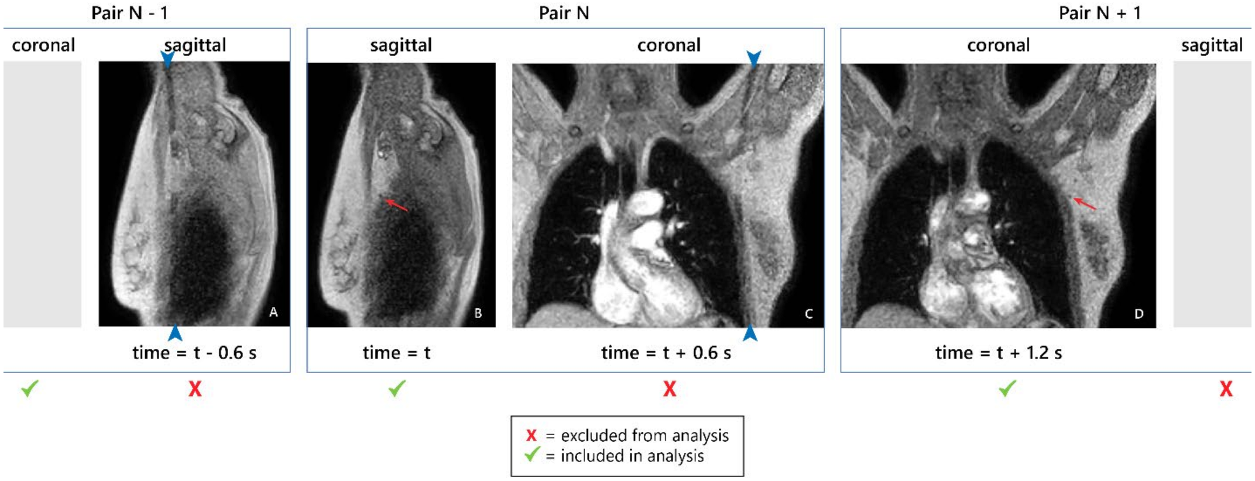

Figure S1. Coronal-sagittal interleaved cine MRI with a small lymph node.

Supplementary Material B – Video results of motion traces shown in figure 2

The videos show the cine MRI and resulting motion traces of the examples shown in figure 2.

Video S1. Motion trace of patient A in supine position (A1 in figure 2).

Video S2. Motion trace of patient A in prone position (A2 in figure 2).

Video S3. Motion trace of patient B.

Video S4. Motion trace of patient C.
